# Critical roles of m^6^A methylation in cardiovascular diseases

**DOI:** 10.3389/fcvm.2023.1187514

**Published:** 2023-05-19

**Authors:** Xinmin Zhang, He Cai, He Xu, Su Dong, Haichun Ma

**Affiliations:** ^1^ Department of Anesthesiology, The First Hospital of Jilin University, Changchun, China; ^2^ The Public Laboratory Platform of the First Hospital of Jilin University, Changchun, China; ^3^ The Cardiovascular Center, The First Hospital of Jilin University, Changchun, China; ^4^ Department of Integrative Medicine, Lequn Branch, The First Hospital of Jilin University, Changchun, China

**Keywords:** cardiovascular diseases, m^6^A methylation, epigenetic, cardiac hypertrophy, heart failure, ischemic heart disease, m^6^A methylation

## Abstract

Cardiovascular diseases (CVDs) have been established as a major cause of mortality globally. However, the exact pathogenesis remains obscure. N6-methyladenosine (m^6^A) methylation is the most common epigenetic modification on mRNAs regulated by methyltransferase complexes (writers), demethylase transferases (erasers) and binding proteins (readers). It is now understood that m^6^A is a major player in physiological and pathological cardiac processes. m^6^A methylation are potentially involved in many mechanisms, for instance, regulation of calcium homeostasis, endothelial function, different forms of cell death, autophagy, endoplasmic reticulum stress, macrophage response and inflammation. In this review, we will summarize the molecular functions of m^6^A enzymes. We mainly focus on m^6^A-associated mechanisms and functions in CVDs, especially in heart failure and ischemia heart disease. We will also discuss the potential application and clinical transformation of m^6^A modification.

## 1. Introduction

Cardiovascular disease (CVD) is a leading major cause of death, responsible for 31.5% of mortalities globally (1). Current evidence suggests that China and India have the highest burdens of CVD worldwide (2).

The past decade has witnessed significant progress achieved in research on CVD, which has led to the development of new therapeutic approaches, such as medications (e.g., angiotensin-converting enzyme inhibitors (ACEIs), angiotensin receptor blockers (ARBs), and statins) and interventional methods [e.g., coronary artery bypass surgery (CABG) and percutaneous coronary intervention (PCI)]. However, current preventive and therapeutic options for CVD remain limited.

With significant inroads achieved in science and technology over the past couple of years, investigations on diseases have progressed to the genetic and epigenetic levels (3, 4). Most of the traditional treatments for patients with CVD are protein-targeting drugs. It has been established that mRNA is subject to tight regulation at the transcriptional and post-transcriptional levels before translation into proteins. Therefore, post-transcriptional targeting has huge prospects for drug development. Above 150 post-transcriptional modifications have hitherto been documented in RNAs in living organisms. A frequent RNA epigenetic modification at the post-transcriptional stage is observed at the N6 position of adenosine, which undergoes N6-m^6^A RNA methylation. Although Desrosiers et al. (5, 6) first reported m^6^A in the 1970s, it is only recently that the mechanisms underlying the specificity of m^6^A modification and biogenesis in cells have been uncovered. m^6^A RNA methylation usually occurs at the RRm^6^ACH consensus motif, which is enriched in internal long exons and 3′ untranslated regions (3′UTRs) near stop codons. Furthermore, m^6^A occurs in precursor mRNAs (pre-RNAs) and long noncoding RNAs (lncRNAs).

In recent years, N6-methyladenosine methylation has been associated with important processes in mammals, such as embryonic development (7), sex determination (8), circadian rhythm (9), neurogenesis (10), stress responses (11) and cancers (12). The functions of m^6^A methylation in cardiovascular diseases have been recognized (13, 14), however, further studies are warranted.

Herein, we discuss the m^6^A proteins respectively. Secondly, we provide a comprehensive overview of the roles of m^6^A methylation in CVDs, laying emphasis on cardiac hypertrophy, heart failure and ischemic heart disease (IHD). Also encompassing atherosclerosis, aortic dissecting aneurysm (ADA), aortic valve calcification, hypoxic pulmonary hypertension (HPH), dilated cardiomyopathy (DCM) and cardiotoxicity. Finally, we discuss future research directions of the application of m^6^A for CVD treatment.

## 2. m^6^A RNA methylation

**Figure 1:**
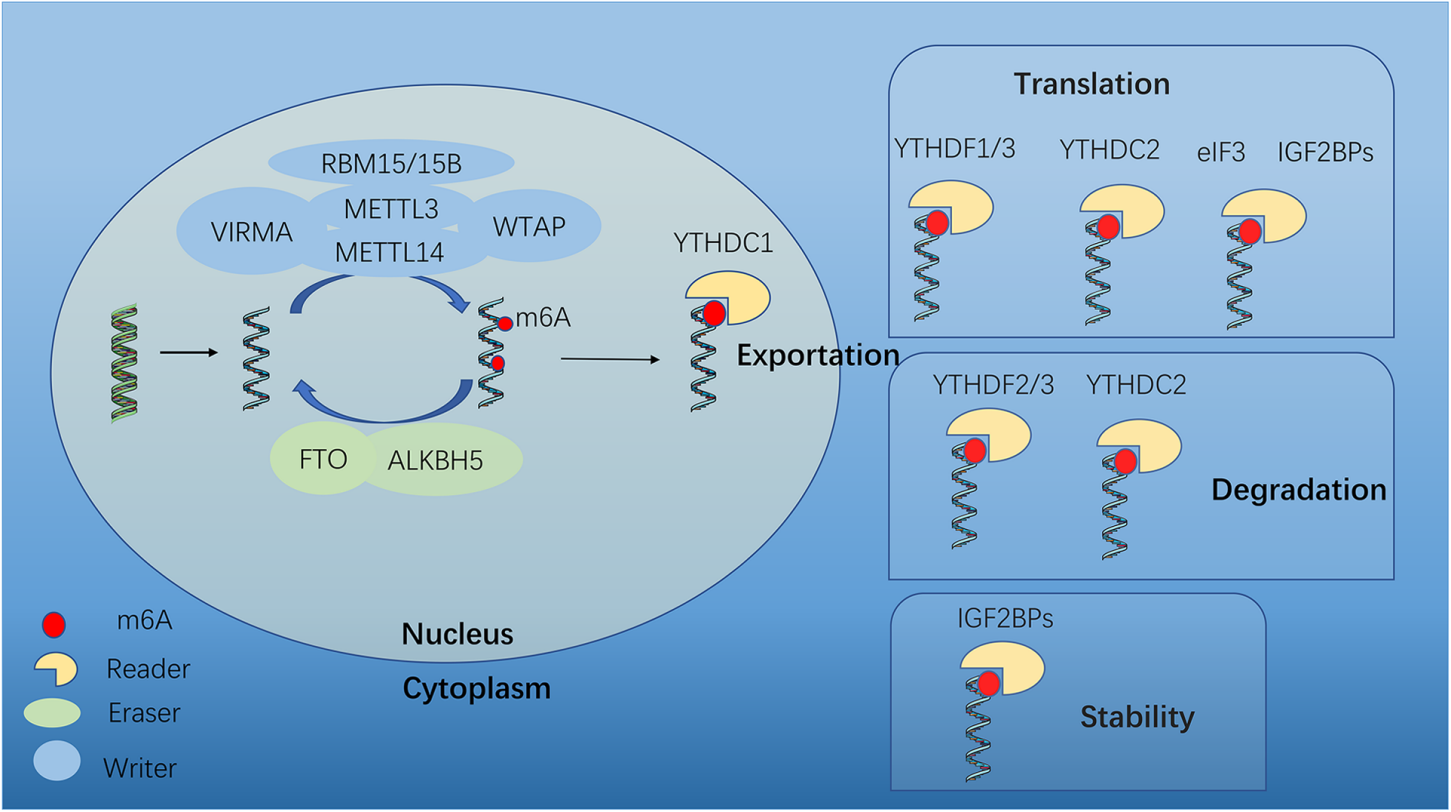
Dynamic m^6^A modification and mediated functions. m^6^A mRNA methylation is regulated by methyltransferases (“writers”), demethylases (“erasers”) and m^6^A-binding proteins (“readers”). METTL3, methyltransferase-like 3; METTL14, methyltransferase-like 14; WTAP, Wilms tumor 1- associated protein; VIRMA, KIAA1429; METTL16, methyltransferase-like 16; RBM15, RNA binding motif protein 15; FTO, fat mass and obesity-associated; ALKBH5, AlkB homologue 5; YTHDF1/2/3, YTH N6-methyladenosine RNA binding protein 1/2/3; YTHDC1, YTH domain containing 1; YTHDC2, YTH domain containing 2; eIF3, Eukaryotic translation initiation factor 3 subunit A; IGF2BP2, insulin-like growth factor 2 mRNA binding protein 2.

The m^6^A modification process involves methyltransferase complexes (writers), demethylase transferases (erasers), and binding proteins (readers) (15) (Figure 1). m^6^A modification can be catalyzed by “writers” and removed by “erasers”. Thus this methylation-dependent process can be reversed and controlled. “Readers” can specifically identify and link RNA methylation modification sites to perform specific biological functions (16), such as RNA splicing, translation and stability (Figure 1).

### 2.1. Methyltransferases/writers

The m^6^A methyltransferase complex is comprised of Methyltransferase-like 3 and 14 (METTL3 and METTL14), which form a stable heterodimer to serve as the catalytic core. Although both METTL3 and METTL14 contain a methyltransferase domain, crystal structure analysis shows that only METTL3 functions as catalytic core, while METTL14 serves as an allosteric adapter to stabilize interaction with RNA binding and improve methylation efficiency. Several auxiliary cofactors facilitate m^6^A deposition as adaptor proteins, by interacting with the core to guide methylase specificity, localization, binding, and activity. These cofactors include Wilms' tumor 1-associating protein (WTAP), VRIMA (KIAA1429), RBM15 (RNA-binding motif protein 15) and ZC3H13. WTAP helps recruit and anchor the methylases to target RNAs, interacts with both METTL3 and METTL14, and guides the specificity and levels of methylation. Methyltransferase-like 16 (METTL16), as a newly identified m^6^A methyltransferase, is the homolog of METTL3 (17). Besides, VIRMA, RBM15 and ZC3H13 are required for m^6^A methylation.

### 2.2. Demethylases/erasers

The m^6^A modification can be removed by “erasers”, including fat mass and obesity-associated (FTO) and AlkB Homolog 5 (ALKBH5). FTO is predominantly expressed in the nucleus, suggesting that any demethylation will occur before mRNA export, and even suggesting that FTO prevents m^6^A addition rather than actively erasing the mark. It has been observed that FTO demethylation in the cytoplasm during cancerous states, DNA damage responses and heat shock, indicating that this may be a specialized pathway. The second known demethylase ALKBH5 was discovered in 2013 (18), which is more likely to serve as a specific eraser of m^6^A, because the expression of ALKBH5 consistently correlates with reduced methylation both in human tissues and mice. Similar to FTO, ALKBH5 exhibits nuclear localization, indicating that cytoplasmic demethylation is largely nonexistent.

### 2.3. Readers

Reader proteins can recognize and bind to the mark, thus paly different downstream effects. They employ effectors to specify transcript splicing, processing, stability, translation, and localization (19). YT521-B homology (YTH) domain containing proteins bind to methyl moiety on the RNA molecule directly and mediate methylated transcript regulation, including YTH domain family proteins (YTHDF1, YTHDF2 and YTHDF3), YTH domain containing 1 and 2 (YTHDC1 and YTHDC2). Other m^6^A regulators bind indirectly, by weaking binding to m^6^A or via m^6^A structural switches, such as eukaryotic translation initiation factor 3H (Eif3), insulin-like growth factor 2 mRNA-binding proteins (IGF2BPs) and heterogeneous nuclear ribonucleoproteins (HNRNPs) (20, 21).

## 3. Cardiac hypertrophy and heart failure (HF)

### 3.1. Pathological hypertrophy and heart failure

**Figure 2:**
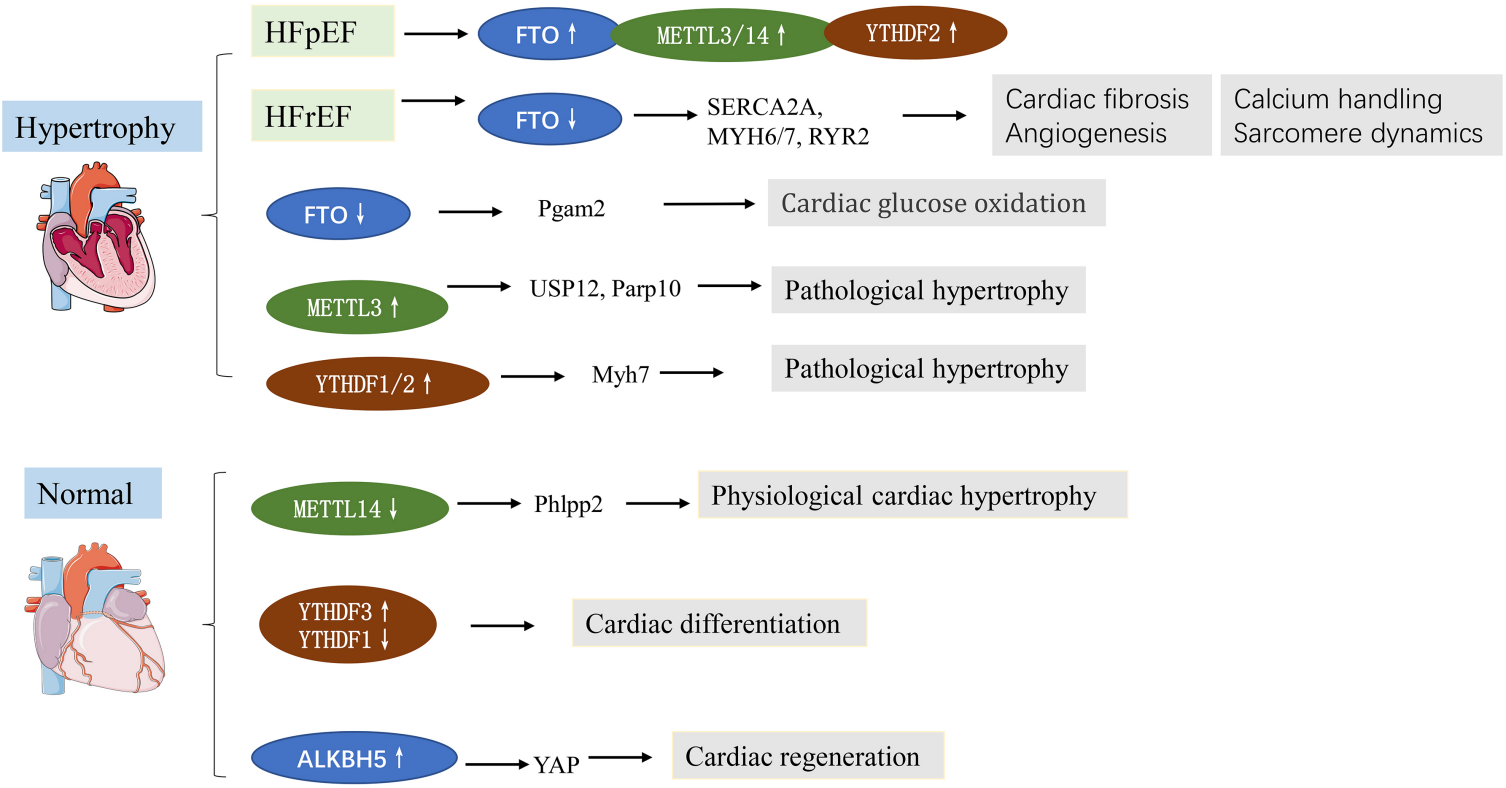
m^6^A methylation in cardiac hypertrophy and heart failure (HF). See main text for more details.

Heart failure represents the terminal stage of various cardiovascular diseases, featuring poor cardiac performance and left ventricular dilatation. Besides, pathological cardiac hypertrophy often results in HF. Clinically, HF is classified into two major subtypes: HF with preserved ejection fraction (HfpEF, EF ≥ 50%) and HF with reduced ejection fraction (HfrEF, EF ≤ 40%). The mechanisms underlying HFpEF are largely obscure. Recently, m^6^A has been closely associated with heart failure in many studies. In this respect, it has been reported that FTO was upregulated in HFpEF patients and mice (22), while FTO was downregulated in HfrEF (13). This differential expression might be caused by pathophysiological differences. Mathiyalagan et al. (13) demonstrated that FTO was downregulated in failing mammalian hearts and hypoxic primary cardiomyocyte (CM) cells. Overexpression of FTO can reportedly increase the m^6^A level in failing hearts and improve cardiac contractile function, as indicated by higher ejection fraction, fractional shortening, and improved wall motion. Similarly, Berulava et al.'s study showed that m^6^A methylation levels were altered in the myocardium of HF and hypertrophy. In cardiac-specific FTO knockout mice, heart failure progression was accelerated, ejection fraction was decreased, and cardiac dilation was increased. METTL3 and FTO knockout may impair the myocardial response to stress load (23). Another study based on a transverse aortic constriction (TAC) model consistently showed that the m^6^A expression gradually increased with time. FTO is the main contributor to increased m^6^A levels. FTO plays a predominant role in increased m^6^A levels. FTO expression was significantly reduced at 8 weeks after TAC, and minimal changes were observed at 1, 3, and 7 days after TAC. FTO overexpression could attenuate cardiac hypertrophy and remodeling and improve cardiac dysfunction and stamina compared with TAC mice (24). Furthermore, FTO plays an important role in cardiomyocyte metabolic homeostasis; the loss of function of FTO has been reported to reduce the glycolytic capacity of cardiomyocytes (24). Mechanistically, FTO targets SERCA2a (a contractile protein) and demethylates SERCA2a resulting in increased SERCA2a expression and cardiac function improvement in mice failing hearts (13) (Table 1). Erkens et al. (52) found that SERCA2a was downregulated in Nrf2 KO mice, associated with LV dysfunction and cardiac hypertrophy, while FTO demethylates SERCA2a, suggesting that FTO is a key factor in Nrf2-associated cardiac hypertrophy. As for cardiac energy metabolism, PGAM2 is involved to a certain extent in the glycolytic changes *in vitro* and *in vivo*. Thus, FTO regulates glycolysis in an m^6^A-dependent way while regulating glucose uptake, possibly by modulating the AKT–GLUT4 axis (24). METTL3, as a “writer” protein, yields an opposite effect compared with “erasers” on cardiac hypertrophy in an m^6^A-dependent manner. Dorn et al. (14) demonstrated that METTL3 expression was increased in hypertrophic cardiomyocytes. Significant m^6^A modification was observed in genes associated with protein kinase mRNAs and intracellular signaling pathways, including members of the MAPK signaling cascade. METTL3 knockout myocardium showed morphological and functional changes of heart failure. Inhibition of METTL3 was sufficient to block hypertrophy *in vitro*, while enhancing METTL3 expression could induce cardiomyocyte hypertrophy without additional stimuli *in vitro* and *in vivo* (14). Kmietzyk et al. (35) found that METTL3 overexpression reduced pathological cardiac hypertrophy, myocardial fibrosis and collagen transcription. FTO knockout attenuated cardiomyocyte hypertrophy in phenylephrine-stimulated cardiomyocyte hypertrophy, while METTL3 knockout increased cell size. These studies overlap in their assertion that METTL3 plays an important role in supporting cardiac homeostasis and hypertrophic stress responses in mice. A subsequent study by Lu et al. (36) validated the effects of METTL3 overexpression on myocardial hypertrophy. A post-translational process termed ubiquitination has been reported to mediate protein stability, intracellular trafficking, and enzyme activity. Lu et al. revealed that USP12 promoted Ang II-induced cardiomyocyte hypertrophy; METTL3 expression was induced by Ang II but was downregulated in USP12 knockdown neonatal rat cardiomyocytes (NRCMs). Upregulation of METTL3 reversed the decrease in myocardial hypertrophy induced by AngII in USP12-silenced NRCMs. Similarly, METTL3 was interacted with Parp10 mRNA, and participate in the prohypertrophic effect of CHAPIR (53). Xu et al. (54) found that the m^6^A reader YTHDF2 was increased in both human and mice HF samples. Furthermore, YTHDF2 suppressed cardiac hypertrophy via m^6^A-mediated degradation of Myh7 mRNA. These studies indicate the potential roles of m^6^A in pathological cardiac hypertrophy and HF (Figure 2).

**Table 1:** Roles of m^6^A enzymes in the cardiovascular system.

Cardiovascular disease	Effector	Expression	Targeted genes	Ref.
**IHD**				
	METTL3	Upregulation	primary miR-143; TFEB; DGCR8; Smad2/3; lncRNAH19	(25–29)
	WTAP	Upregulation	ATF4	(30)
	METTL4	Upregulation	Wnt1; lncRNAH19	(28, 31)
	FTO	Downregulation	SERCA2a	(32)
	METTL3	Downregulation	Bax and PTEN	(33)
**Cardiac hypertrophy and HF**				
HF	FTO	Downregulation	SERCA2A, MYH6/7, RYR2; Mhrt; FOXO1, FOXO4, ELF2,EIF5a; SMYD1, DICER1, RBM20; ERK,MDM2; Pgam2	(13, 23, 24, 34)
Pathological hypertrophy	METTL3	Upregulation	Arhgef3, Myl2; MAP3K6, MAP4K5, MAPK14; P300; miR-221/222	(14, 35–37)
Physiological hypertrophy	METTL14	Downregulation	Phlpp2	(38)
**Hypoxic stress myocardium**				
	ALKBH5	Upregulation	WNT5A	(39)
	METTL3	Upregulation	NCBP3	(40)
**ADA**				
	FTO	Upregulation	Klf5	(41)
	METTL14	Downregulation	No mention	(42)
**Aortic valve calcification**				
	METTL3	Upregulation	TWIST1	(43)
**Myocardial inflammation and sepsis**				
	FTO	Downregulation	IL-6 and TNF-α	(44)
**Atherosclerosis**				
	METTL14	Upregulation	FOXO1; Myd88	(6, 45)
	METTL3	Downregulation	EGFR	(46)
**Cardiomyopathy**				
Hyperlipidemia-induced Cardiomyopathy	FTO	Upregulation	CD36	(47)
DCM	YTHDC1	Downregulation	Titin	(48)
	FTO	Downregulation	Mef2a, Klf15, Bcl2l2, Cd36, and Slc25a33	(49)
**Hypoxic pulmonary arterial hypertension**				
	METTL3	Upregulation	PTEN	(50)
	METTL14	Upregulation	SETD2	(51)

IHD, ischemia heart disease; HF, heart failure; ADA, aortic dissecting aneurysm; DCM, dilated cardiomyopathy.

### 3.2. Physiological cardiac growth and regeneration

Loss of cardiomyocytes following cardiac injuries plays a key role in the development of heart failure. In mammals, cardiomyocytes have long been considered as permanent cells with no ability to proliferate. However, recent studies show that, in fact, cardiomyocytes in mammals have some degree of regenerative capacity during development and very soon after birth (55). m^6^A is involved in cardiomyocyte proliferation and differentiation (56). A study showed that m^6^A modification and m^6^A peaks were lowest in 1-day-old mouse hearts than at 7 and 28 days after birth (57). Zhenbo Han et al. (58) investigated the importance of m^6^A alteration in heart regeneration during postnatal and adult injury. As expected, m^6^A demethylase ALKBH5 was downregulated, while the global m^6^A level was increased after birth. Cardiac function and regeneration ability decreased significantly in ALKBH5 knockout mice after neonatal apex resection. After induction of ALKBH5 expression, the myocardial infarction area was significantly reduced, cardiac function was restored, and CM proliferation was promoted after myocardial infarction in young and adult mice. ALKBH5 promoted YAP translation by increasing YTHDF1 levels. Interestingly, two similar proteins, YTHDF1 and YTHDF3, reportedly yield distinct effects on the same biological process (59). Recent evidence revealed that although YTHDF1 and YTHDF3 play diverse roles in embryonic stem cell-derived cardiac differentiation, they exhibit decreased levels. Loss of YTHDF1 could downregulate cardiomyocyte-specific genes and impair their differentiation. In contrast, YTHDF3 knockdown promoted differentiation by upregulating CM-specific genes (59).

Multiple studies have demonstrated that the endogenous regenerative potential of cardiomyocytes in the adult heart can be activated by interventions like exercise, and exercise can induce physiological cardiac hypertrophy in the heart. Although pathological cardiac hypertrophy and physiological cardiac hypertrophy appear similar, the underlying mechanisms that exist are fundamentally different. m^6^A is reportedly essential for exercise-induced physiological cardiac hypertrophy (38). METTL14 is downregulated during exercise-induced physiological cardiac hypertrophy, while METTL14 knockdown impairs cardiac dysfunction during ischemia-reperfusion remodeling. Mechanistically, METTL14 mediates cardiomyocyte development and apoptosis by suppressing Phlpp2 mRNA m^6^A modifications and activates Akt-S473. microRNAs are important in both cardiac pathologies and physiologies. microRNA-222 (miR-222) was upregulated in exercise-induced physiological cardiac hypertrophy, protecting heart against adverse remodeling (60). It has been reported METTL3 directly regulate miR-221/222 by promoting miR-221/222 maturation in Ang-II-induced cardiac hypertrophy, subsequently activating the Wnt/*β*-catenin pathway (37). Overall, these studies indicate m^6^A is essential in preserving cardiac homeostasis and associated with cardiac regeneration.

The above findings support the important roles of m^6^A modification in cardiac hypertrophy (physiological and pathological) and heart failure, highlighting that it is a promising therapeutic strategy for the diagnosis and therapy of HF. In addition, finding the targets of physiological cardiac hypertrophy may be of great significance for the treatment of pathological cardiac hypertrophy, and the m^6^A-dependant way may be an important target (Figure 2).

## 4. Ischemic heart disease

### 4.1. Myocardial infarction (MI)

IHD represents a significant threat to public health worldwide. Gong et al. (25) demonstrated that myocardial infarction was decreased and cardiac function improved in METTL3 knockout mice, consistent with findings reported by Song et al. (26), who demonstrated that METTL3 is a negative regulator of autophagy in cardiomyocytes; however, ALKBH5 has the opposite effect. There is an increasing consensus that METTL3 and METTL14 are increased in hypoxic and reoxygenated(H/R) cardiomyocytes and IR myocardium (26, 31, 40). The upregulation of METTL3 was found to suppress autophagic flux and potentiate apoptosis in H/R-treated cardiomyocytes. TFEB is a key downstream target gene of METTL3, and TFEB mRNA expression decreases after METTL3 overexpression. Overexpression of TFEB or ALKBH5 reversed the effect of METTL3 on H/R cardiomyocytes. In addition, METTL3 interacted with NCBP3, facilitating the translational process in the myocardium under hypoxia stress (40). Thus, METTL3-mediated m^6^A modification represents a pivotal hypoxic stress response.

m^6^A level is increased in fibrotic tissues after an MI and in TGF-*β*1-treated CFs. METTL3 is the most significantly altered protein. It has been reported that METTL3 overexpression activated CF deposition and promoted collagen synthesis and deposition, while its inhibition improved myocardial fibrosis and cardiac function (27).

Unlike retina angiogenesis (61), no METTL3 upregulation has been observed in hypoxic cardiac microvascular endothelial cells (CMECs) (39). In contrast, significant ALKBH5 upregulation was observed in hypoxic CMECs, which impaired their proliferation, migration, and tube formation, while m^6^A levels were decreased. ALKBH5 knockdown increased angiogenic phenotypes in hypoxic but not in normoxic CMECs. ALKBH5 regulated postischemic angiogenesis by post-transcriptional modulating and destabilizing WNT5A mRNA in an m^6^A-dependent manner (39). In addition, sympathetic hyperactivity after myocardial infarction is related to METTL3 (62), which supports the old concept of the brain-heart axis in neurocardiology (63).

### 4.2. Myocardial ischemia-reperfusion (MI/R)

Timely revascularization is the standard care treatment of IHD, but the recovery of blood flow after myocardial ischemia can cause further tissue damage [myocardial ischemia-reperfusion (MI/R) injury] (64). An increasing body of evidence from recently published studies suggests that m^6^A modification is present in many important pathological processes, such as cell death (apoptosis, pyroptosis), autophagy, and endoplasmic reticulum stress, which have played significant roles on MI/R and were validated by bioinformatics analysis (65).

Oxidative stress and apoptosis are important pathological processes in ischemia-reperfusion injury (IRI) (66). The pathophysiological processes can be fine-tuning via epigenetic post-transcriptional modifications, such as m^6^A methylation, by regulating post-transcriptional RNA levels. METTL14, as a “writer” protein, is upregulated during ischemia-reperfusion and oxidative stress-induced cardiomyocyte injury. METTL14 deficiency aggravates myocardial injury and dysfunction. It has been reported that METTL14 overexpression significantly alleviated infarct size and apoptosis and improved cardiac function during I/R injury. Further study found that METTL14 activated the Wnt/*β*-catenin signaling pathways through methylating Wnt1 mRNA. Wnt1 knockout eliminated the METTL14-mediated protective effect against myocardial injury and apoptosis (31). Shen et al. (34) demonstrated that FTO inhibits H/R cardiomyocyte apoptosis by regulating Mhrt mRNA. Hypoxic preconditioning/ischemic preconditioning(HPC/IPC) is reportedly protective against myocardial ischemia/reperfusion (MIRI) (67). H19, an imprinted lncRNA, participates in MIRI and cardiomyocyte hypertrophy (68, 69). Y. Su et al. (28) demonstrated that METTL3 and METTL14 interact with lncRNA H19 to reduce H9c2 cell apoptosis, highlighting their importance in HPC treatment.

Current evidence suggests that during myocardial IRI, endoplasmic reticulum (ER) stress is important in I/R-induced damage mediated by m^6^A. Wang et al. (30) demonstrated that WTAP targeted activating transcription factor 4 (ATF4), one of the stress-responsive transcription factors, by activating ATF4 mRNA stability. Mechanistically, WTAP knockdown downregulated ATF4 mRNA stability and protected cardiomyocytes against apoptosis and ER stress. In contrast, overexpression of WTAP induced apoptosis and ER stress.

Pyroptosis is involved in many pathological processes, including MI/R injury, METTL3 aggravated cardiomyocyte pyroptosis through promoting DGCR8 binding to pri-miR-143-3p, thus inhibiting PRKCE transcripton (29).

**Figure 3:**
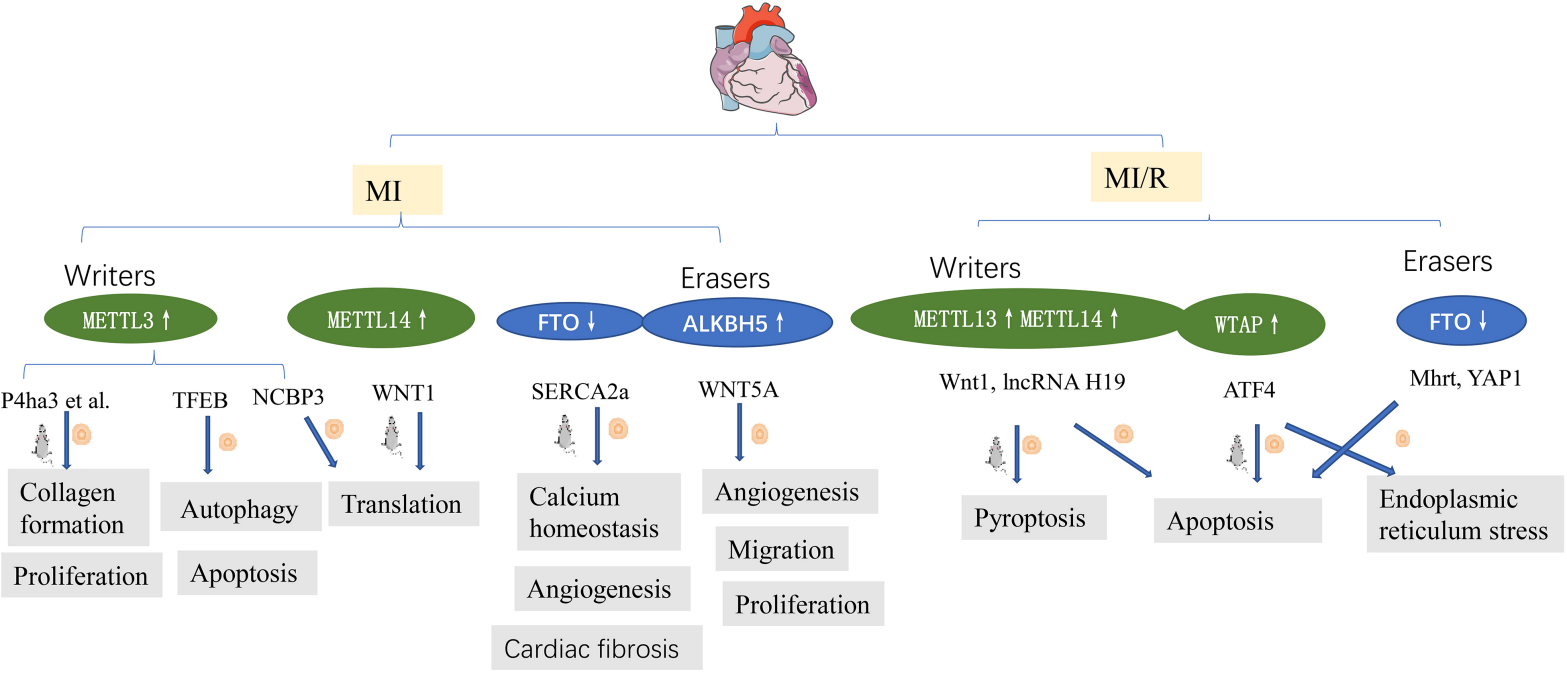
m^6^A methylation in ischemia heart disease. See main text for more details.

Xuan Su found that the global level of m^6^A methylation and METTL3 protein were down-regulated both in young and elderly hearts after I/R injury, while FTO was only decreased in aging myocardium (33). In line with this, *in vitro* studies revealed that FTO was decreased in hypoxic cardiomyocytes (13, 70). Overexpression FTO reversed MI-induced high levels of m^6^A, reduced the myocardial infarction area and the degree of fibrosis, and enhanced angiogenesis (13). FTO targets SERCA2a and plays an important role in calcium homeostasis, enhancing the energy metabolism of H/R cardiomyocytes and cardiac contraction (13, 32). FTO enhanced the stability of YAP1 mRNA in cardiomyocytes following H/R injury by disrupting the m^6^A modification of YAP1 mRNA (70) (Figure 3). myocardial fibrosis was relieved after FTO overexpression. It has been confirmed the interaction between FTO demethylates and a series of mRNAs, such as Mef2a, Klf15, Bcl2l2, Cd36, Slc25a33 (49).

Overall, m^6^A expression in myocardial ischemia-reperfusion injury is complex. Similar proteins often have different functions in the heart and exhibit significant heterogeneity in distribution with age and body part. Even the distribution of m^6^A proteins varied in different parts of the heart (71). Further research is warranted to uncover the different functions of m^6^A modifications in MI/R.

## 5. Aortic dissecting aneurysm (ADA)

It is widely acknowledged that ADA features an intimal flap separating the true and false lumens (72). Growing evidence suggests that the m^6^A methylation is significantly altered in ADA tissue (41, 42), although significant inconsistencies have been reported in the literature.

In Ma et al.'s study (41), FTO expression was significantly upregulated in human aortic dissection (AD) tissues compared to the aortic aneurysm (AA) group. Another study reported significantly decreased FTO expression in AD tissue samples compared with normal samples, while METTL14 was significantly upregulated (42).

Current evidence suggests that forced expression of FTO potentiates vascular smooth muscle cell proliferation and migration and upregulation of its target gene, Klf5 (41). m^6^A levels play a determining role in the abdominal aortic aneurysm (AAA) and ADA (73), regulating the metabolism and stability of mRNA, suggesting that m^6^A represents a potential target to prevent aortic diseases.

## 6. Aortic valve calcification (AVC)

Aortic valve calcification is one of the most common cardiac valvulopathies. Current evidence suggests that human aortic valve interstitial cells (hVICs) are predominantly found in the aortic valve. It has been shown that METTL3 is upregulated, and twist-related protein 1 (TWIST1) is downregulated in AVC. Luciferase reporter assays and MeRIP-qRT-PCR confirmed the interaction between METTL3 and TWIST1. Further study showed that METTL3 inhibited TWIST1 and promoted osteogenic differentiation of human aortic valve interstitial cells via an m^6^A-YTHDF2-dependent pathway (43).

## 7. Inflammation and sepsis

Sepsis is an organ dysfunction-related systemic inflammatory response to infection associated with high morbidity and mortality rates globally (74). Cardiovascular dysfunction attributed to sepsis was first documented in 1951 by Waisbren (75). A higher mortality rate has been observed in sepsis patients presenting with cardiovascular dysfunction than those without (76). Although many pathways and mediators have been associated with myocardial depression in sepsis, the exact cause remains obscure (77).

A study on sepsis-induced myocardial dysfunction revealed that in septic heart tissues, the global m^6^A levels were significantly decreased; The changes of the m^6^A modification levels were significantly in mRNAs and lncRNAs. Pathway analyses revealed significant enrichment in immune and inflammatory response pathways (78). In contrast, Dubey et al.'s research showed increased m^6^A modification with downregulated FTO in LPS-induced myocardium *in vitro* and *in vivo* (44). Although the two studies yielded contrasting findings, m^6^A modification remains crucial in inflammatory signaling pathways of the sepsis myocardial injury model. The genes of inflammatory cytokines (IL-6, TNF-α, IL-1β) were upregulated, and left ventricular function was reduced in the sepsis hearts (44). Feng et al. found that by mediating the alternative splicing of MyD88, METTL3 could inhibit the inflammatory response triggered by lipopolysaccharides (79). In another study by Jian et al. (6), METTL14 expression but not METTL3 expression was significantly increased in endothelial cell inflammation induced by TNF-α, suggesting that METTL3 and METTL14 play different regulatory roles in m^6^A modification although they work synergistically. Macrophages represent a vital immune system component and are crucial in the inflammatory process. Rui Yu et al. revealed upregulation of YTHDF2 during the LPS-induced inflammatory response of macrophages (80). YTHDF2 knockdown promotes the release of proinflammatory cytokines and exacerbates inflammation in LPS-stimulated RAW 264.7 cells by activating MAPK and NF-*κ*B signaling pathways.

Overall, m^6^A modification represents a potential target to attenuate cardiac inflammation and dysfunction during endotoxemia or sepsis.

## 8. Atherosclerosis

Atherosclerosis is the dominant cause of cardiovascular diseases, characterized by lipid accumulation in the walls of arteries (81). Overwhelming literature substantiates atherosclerosis as a chronic inflammatory disease associated with lipid accumulation (82, 83). There is a rich literature available substantiating that epigenetic processes, including DNA methylation, histone modification and m^6^A RNA methylation, play an important role in atherosclerosis (6, 46, 47, 84).

Growth factor receptors (EGFR) can accelerate the formation of atherosclerotic lesions (85). It has been reported that METTL3 is decreased in atherosclerosis regions. Further studies demonstrated that m^6^A modification of the EGFR mRNA 3′UTR contributes to atherogenesis (46). A mechanistic analysis revealed that m^6^A could interact with the EGFR mRNA; m^6^A modification of the EGFR 3′UTR accelerated its mRNA degradation leading to endothelial dysfunction. Moreover, METTL3 overexpression significantly reduced EGFR activation and endothelial dysfunction during oscillatory stress (OS). Thrombospondin-1 (TSP-1), a shear-sensitive protein, is vital in regulating vascular remodeling. Interestingly, TSP-1/EGFR inhibition using shRNA and AG1478 prevented atherosclerosis development. These results suggest that METTL3 and m^6^A modifications could alleviate endothelial activation and atherogenesis by accelerating the degradation of oscillatory flow-induced EGFR mRNA expression (46). Jian et al. (6) found that downregulated METTL14 expression could suppress endothelial inflammation and atherosclerotic progression. Furthermore, *in vivo* experiments were carried out in METTL14 knockout mice. After 12 weeks of western diet (WD) feeding, a significant decrease in lesion size was observed in METTL14 knockout mice. METTL14 interacted with FOXO1 enhancing its translation by increasing m^6^A modification, thus upregulating the expression of adhesion molecules, regulating endothelial monocyte adhesion, and participating in atherosclerosis progression. Similarly, it has been reported that the knockdown of METTL14 mitigates the macrophage inflammatory response by promoting M2 polarization via the NF-*κ*B pathway (45). Since METTL3 and METTL14 are “writers”, they should theoretically have similar functions. However, current evidence suggests synergistic m^6^A proteins play diverse roles in different models and pathways.

**Figure 4:**
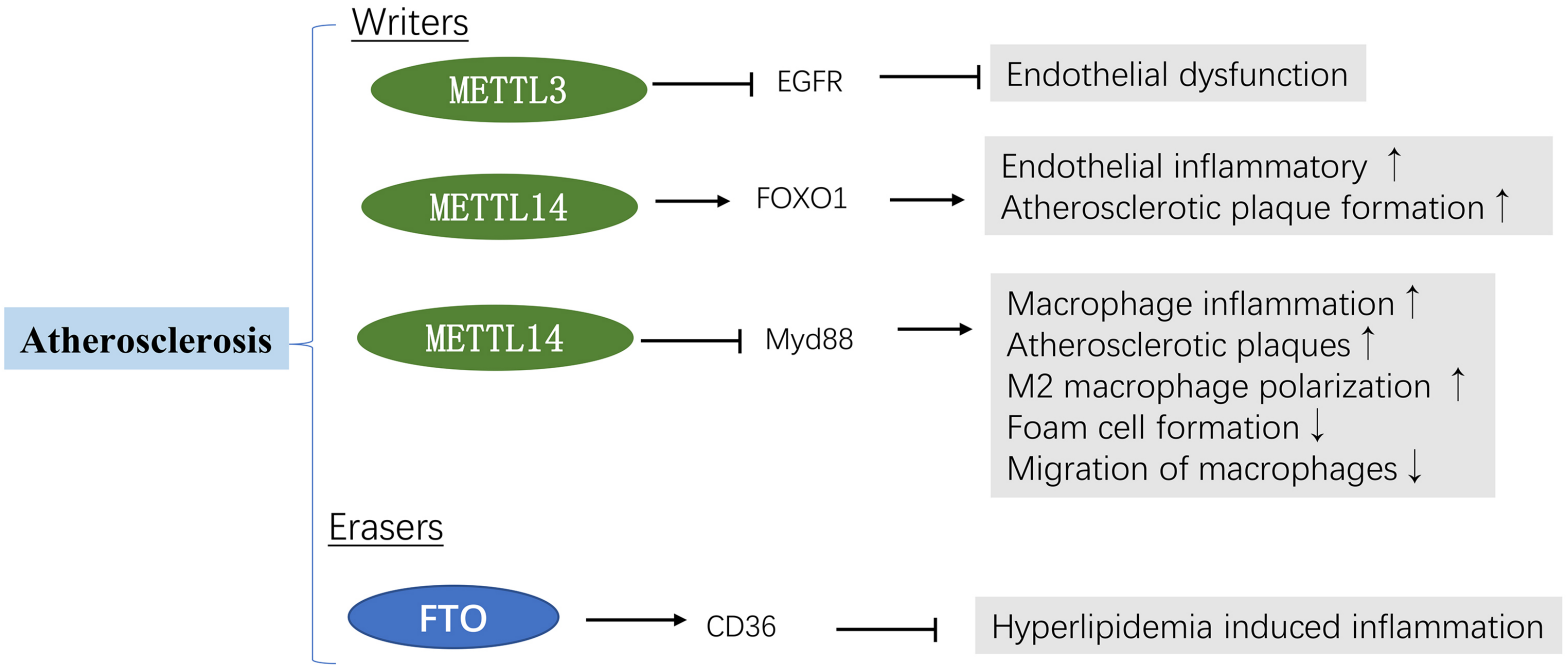
m^6^A methylation in atherosclerosis. See main text for more details.

As mentioned above, lipid accumulation is an important factor in atherosclerosis progression. It is now understood that a high-fat diet (HFD) leads to cardiac lipid deposition in obesity cardiomyopathy. In contrast, intermittent fasting (IF) (*ad libitum* feeding alternated with fasting periods) has been reported to yield cardioprotective effects (86). A study showed that IF could ameliorate the effects of HFD-induced cardiac dysfunction and serum lipid metabolic disorder (87). IF downregulated the mRNA levels of genes associated with fatty acid uptake and synthesis, upregulated fatty acid catabolism genes, and decreased the m^6^A methylation levels (decreased METTL3 expression and increased FTO expression) in HFD-induced obesity cardiomyopathy (87). Consistently, the LuHui Derivative (LHD), a novel synthetic anthraquinone compound, has been reported to reduce lipid deposition in cardiomyocytes (47). Interestingly, the cluster of differentiation 36 (CD36) as a downstream target of LHD participates in treating cardiac inflammation triggered by palmitic acid. LDH can bind to FTO and elevate intracellular m^6^A levels, alleviating hyperlipidemia-induced inflammation in cardiomyocytes. Moreover, FTO overexpression significantly upregulated CD36 expression and inhibited LHD's anti-inflammatory effects. Conversely, silencing FTO inhibited cardiac inflammation induced by palmitic acid by decreasing the stability of CD36 mRNA (47) (Figure 4).

## 9. Hypoxic pulmonary hypertension

HPH is a cardiopulmonary disease featuring increased pulmonary artery pressure and remodeled small pulmonary arteries conducive to right heart failure (88, 89). Epigenetic processes, such as DNA methylation, are pharmacologically reversible, making them an attractive target as therapeutic strategies for pulmonary arterial hypertension (PAH) (90). It is widely thought that m^6^A modifications mediate the development of HPH.

**Figure 5:**
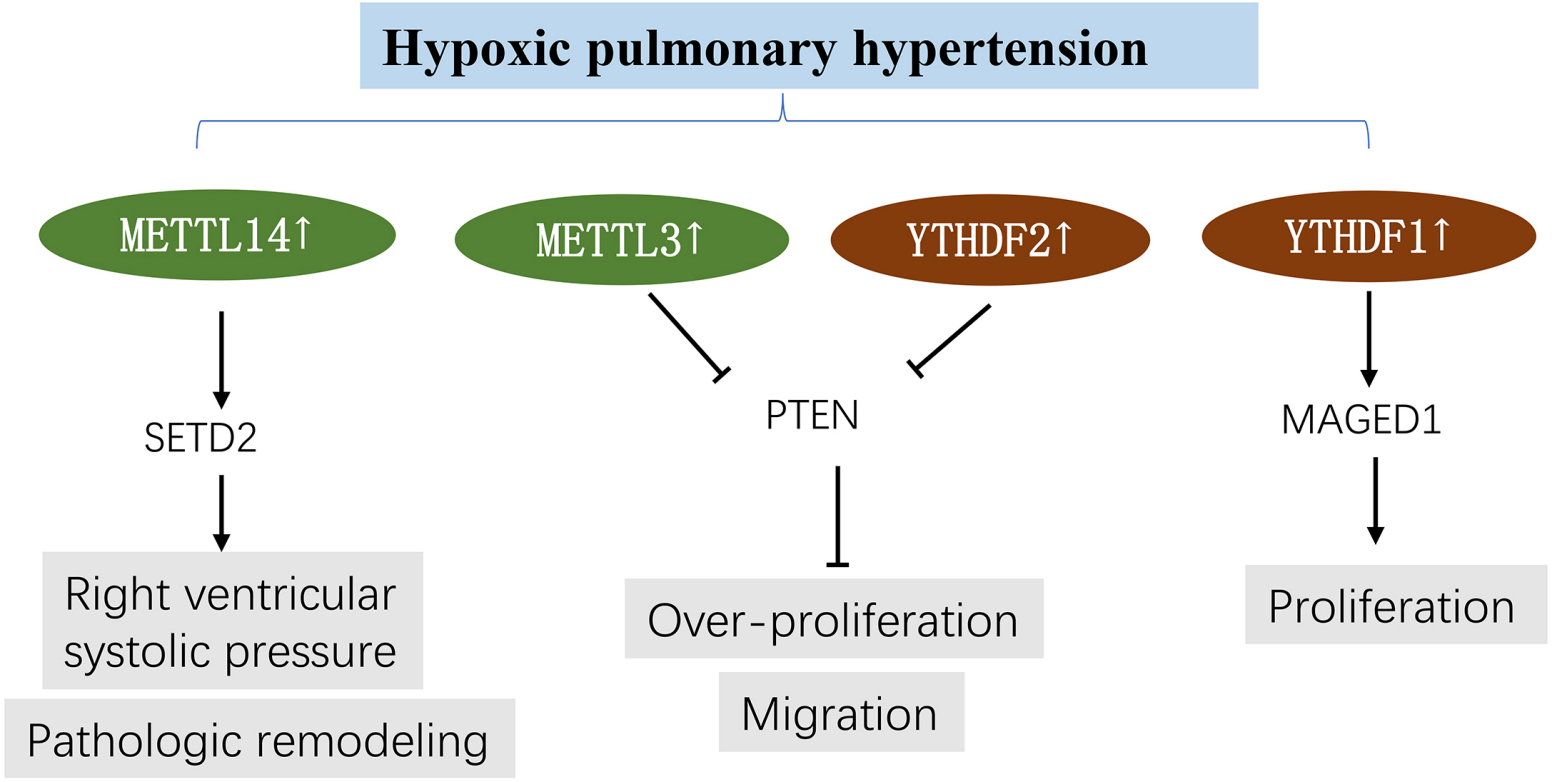
m^6^A methylation in hypoxic pulmonary hypertension. See main text for more details.

Qin et al. (50) and Hu et al. (91) used pulmonary artery smooth muscle cells (PASMCs) and hypoxic rat models to study m^6^A modifications in hypoxic pulmonary hypertension. The results showed that METTL3 and YTHDF2 were highly expressed in hypoxia-induced PASMCs and hypoxic pulmonary arteries (50), similar to YTHDF1 (91). Phosphatase and tensin homologue (PTEN) is reportedly the target gene of METTL3, and its increased degradation has been strongly associated with high YTHDF2 expression. Downregulation of METTL3 prevented PASMC proliferation and migration induced by hypoxia; however, downregulation of PTEN yielded the opposite effects by triggering the PI3K/Akt signaling pathway. The importance of the METTL3/YTHDF2/PTEN axis in HPH has been established (50). Similarly, Hu et al. confirmed that YTHDF1 knockdown could ameliorate proliferation phenotype switch and pulmonary hypertension development by targeting MAGED1 *in vivo* and *in vitro*. YTHDF1 could recognize and promote the translation of MAGED1. In addition, MAGED1 silencing mitigated pulmonary artery smooth muscle cell proliferation induced by hypoxia (91). Most importantly, Zhou et al. (51) found that SETD2 and METTL14 are promising targets in PAH. SETD2 deficiency could alleviate pulmonary arterial pressure and pathologic remodeling and improve right ventricular function and cardiac hypertrophy in hypoxia-induced PAH. Furthermore, silencing SETD2 in SMCs markedly decreased METTL14 and global m^6^A levels in PAH (Figure 5).

With a refined understanding of the epigenetic processes involved in PAH, m^6^A RNA methylation has huge prospects as a target to prevent and treat this patient population.

## 10. Dilated cardiomyopathy (DCM)

DCM is a condition whereby the left ventricle is dilated and associated with systolic dysfunction (92). In DCM, the total m^6^A levels were higher than normal hearts, while FTO protein were downregulated. FTO overexpression improved cardiac function in DCM mice (49). Genetic mutation of sarcomeric genes is an important cause of DCM (92). Although its mechanisms remain elusive, Gao et al. (48) established that YTHDC1 knockdown led to DCM. Current evidence suggests that Titin (TTN) mutations are responsible for 20%–25% of sarcomeric gene mutations, and an increased ratio of N2BA: N2B (two major Titin mRNA isoforms) is conducive to DCM. Dysregulated Titin pre-mRNA splicing results in an uneven N2BA: N2B ratio. Further research indicated that YTHDC1 deficiency causes aberrant splicing of Titin, increasing the ratio of N2BA: N2B isoform, ultimately leading to DCM. Overall, YTHDC1-dependent Titin splicing has huge prospects for treating DCM (48).

## 11. Cardiotoxicity

Cardiotoxicity is a well-known adverse effect of anticancer drugs (93, 94). Acute cardiotoxicity often presents with electrocardiogram (ECG) changes and arrhythmias, which lead to palpitations, presyncope and syncope, and even cardiac arrest. Chronic cardiotoxicity includes ventricular dysfunction, dilated cardiomyopathy, and heart failure (94). Tumor therapy drugs, such as doxorubicin (DOX) and cyclophosphamide (CYP), have raised significant concern, given their cardiotoxicity (95, 96). The past decade has witnessed a burgeoning interest in the molecular pathways of cardiotoxicity, including m^6^A modification.

Zhuang et al. found that ferroptosis is crucial in DOX-induced cardiotoxicity and may be associated with m^6^A RNA modification (95). The long noncoding RNA KCNQ1OT1, a miR-7-5p sponge, is modified by m^6^A through the action of METTL14. Besides, it has been established that miR-7-5p targets METTL14. Such a feed-forward mechanism emphasizes METTL14's crucial contribution to ferroptosis and cardiotoxicity attributed to DOX. Our findings suggest that a novel treatment strategy to manage DOX-induced cardiac injury may involve selectively reducing ferroptosis in cardiomyocytes, which is mediated by a METTL14/KCNQ1OT1/miR-7-5p positive feedback loop. Interestingly, cyclophosphamide has been associated with cardiac electrical and contractile alterations. In addition, cyclophosphamide can reportedly induce RNA m^6^A modification by upregulating METTL3 expression and suppressing JPH2 expression (96). These findings indicate that m^6^A is a potential target for preventing and treating drug-induced cardiotoxicity.

## 12. Conclusion and future perspectives

Epigenetics is a hot scientific research topic in recent years. In this review, we provided a comprehensive overview of the critical roles and mechanisms of m^6^A methylation in CVDs (Table 1). In general, m^6^A exists commonly with significant and complex roles. For example, the role of the same protein is distinct in different cells and exhibits heterogeneous levels in the same tissue (24, 61). In addition, different conclusions are gained from the same research topic, in Ma et al.'s study, FTO expression was elevated in human AD tissues (41), but downregulated in AD tissue in another study (42). Even different levels of METTL3 and METTL14 proteins have been documented in anatomical regions of mouse adult hearts, and these levels were altered with aging (71).

With the rapid development of detection methods and artificial intelligence (AI) are applied in discovery of m^6^A targeting drugs, m^6^A modulators will be the potential and promising therapeutic targets in the future.

To date, many compounds targeting m^6^A methyltransferase and demethylases have been identified, especially in cancers. For example, METTL3 inhibitor is emerging as a new target to treat acute leukemia (97) and so is YTHDF2 inhibitor (98). Paris et al. (99) found a new lead treatment with FTO inhibitor in glioblastoma. FB23-2, an inhibitor of FTO, impairs proliferation and enhances differentiation of acute myeloid leukemia (AML) cells (100).

The cardiovascular regulating roles of m^6^A modulators are relatively fewer than cancers. IOX1, an inhibitor of ALKBH5, was loaded onto ferritin nanocage and it was found to effectively improve cardiac function (101). FTO is overexpressed in human artery specimens of obese individuals, and its inhibitor rhein or FB23-2 exerts protective effect with increasing prostaglandin D2 production and myogenic tone (102). Thus, m^6^A modulators are of translational and therapeutic interests in either cancers or cardiovascular diseases. But only a few of identified regulators are druggable and suitable for treatment. Due to the poor target specificity, pharmacokinetics, therapeutic safety and efficacy (99, 100), none of the regulators has been approved for clinical treatment.

Since m^6^A targeted drugs are widely used in tumors. As mentioned before, tumor drugs will cause cardiotoxicity in the application process. If an m^6^A regulated drug can be developed, it will kill two birds with one stone by acting on tumors while reducing their cardiotoxicity. Moreover, among all the studies of HF and ischemia heart, FTO and ALKBH5 have been proved to be therapeutic and studied most frequently, we speculate that compounds of m^6^A erasers could hold translational therapeutic value to treat ischemic hearts and HF. More experimental and clinical evidence is needed to substantiate the role of m^6^A methylation. Researching and developing targeted drugs for different diseases and various stages of the same disease can be challenging and promising, emphasizing the need for further studies.
